# Irish Local Government Boards and Disloyal Boards of Guardians

**Published:** 1917-01-20

**Authors:** Maurice R. J. Hayes

**Affiliations:** Honorary Secretary. Irish Medical War Committee, c/o The British Medical Association, 16 South Frederick Street, Dublin


### Irish Local Government Boards and Disloyal Boards of Guardians.

*To the Editor of* THE HOSPITAL.

SIR,—My attention has been directed to a paragraph in your issue of December 9, page 197, entitled, "The Irish Local Government Board and Disloyal Boards of Guardians."

I am not concerned with the charges of alleged "active disloyalty to the Allied cause" or to the implied disloyalty of a well-known Irish Unionist M.P., who in his professional capacity has expressed a legal opinion, but the charge of disloyalty which is made against the Irish doctors is so unjust and so baseless that it cannot be allowed to pass unquestioned.

In Dublin the staffs of all our medical schools and hospitals have contributed to the R.A.M.C., and the work of those who have volunteered is being patriotically carried on at the expense of much additional labour by those who have remained at home. Not only is this labour cheerfully borne, but the hospital staffs, in addition to these increased duties, are giving their valued services gratuitously to the Castle and other Red Cross hospitals. This is true also in respect of practitioners in Belfast, Cork, Galway, and throughout Ireland. As evidence of the whole-hearted manner in which the Irish doctors have voluntarily joined the Colours, I may state that in July 1915 it was computed that a certain percentage of the total number of doctors in the different parts of the British Isles was deemed sufficient for the needs of the Army. At this period Ireland had already without effort practically contributed its quota, as is proved by the following official testimony : "As far as we can see, Ireland has practically done what Scotland set out to do." That there has been no diminution in medical recruitment from Ireland is borne out by the following official acknowledgment received on November 10, 1916 : "We should like to take this opportunity of offering our warmest congratulations to your committee on the steady flow of volunteers it is securing."

The task which the Irish Medical War Committee has found easy will not be rendered more easy by ill-informed and venomous attacks on the Irish medical profession such as this.—I am, yours truly,

MAURICE R. J. HAYES.
Honorary Secretary.

Irish Medical War Committee,
c/o The British Medical Association,
16 South Frederick Street, Dublin,
January 10, 1917.

*** 1. There is in the paragraph alluded to no imputation of disloyalty to an Irish M.P. ; the words used will not bear that construction, and were not intended to do so.

2. There is no charge of disloyalty against the Irish medical profession, but only against Ireland as a whole; this is notorious, and we note that the Irish Medical War Committee does not deny it.

3. It is entirely beside the point to compute the relative contributions to the Allied cause of Irish and British doctors in July 1915. At that time the Military Service Act was not passed or proposed. The state of affairs now, in 1917, is that every British doctor between twenty-one and forty-one is serving the State unless his services are deemed indispensable to the civilian population. This is not true of Irish doctors; the case of Letterkenny is not an isolated one, as is well known. Admittedly the Irish medical profession has responded very well to the call for recruits; but the official acknowledgment of this is not proof that Ireland is doing as much as the rest of the kingdom in this respect.

4. It is useless for Irishmen, whatever their politics, to shut their eyes any longer to the fact that hostility and disloyalty to England are also hostility and disloyalty to France, Belgium, Russia, and the other Allies, and also (in the belief of the Allies) to truth, justice, and freedom. The Letterkenny incident and others of the same sort are merely active expressions on the part of Irish local authorities of hostility and disloyalty to England; the fact that the voting on these occasions has been on strictly party lines proves that. If the Irish medical profession is in earnest in its desire to repudiate the disloyal section which exists in it—and we have every confidence that this is the case—let it take such administrative steps as will advertise its aversion from disloyalty. The Irish Local Government Board has set the example, so there is no lack of precedent. Let the Irish Colleges and other educational bodies memorialise the Government in such a way as to strengthen its hands when incidents such as those at Letterkenny and Omagh arise. Let young Irish doctors who will not join the R.A.M.C. be excluded, whenever it can legally be done, from candidature for all hospital and other public appointments, not only now but for all time; and let the Irish Medical War Committee take steps to promote such regulations and such other administrative measures as may promote the same end.

5. THE HOSPITAL has no desire to embarrass recruiting in Ireland, whether for the R.A.M.C. or for any other branch of the Army; but it is useless to blink facts, and the fact is that Ireland is not pulling her weight in the Allied cause. We are happy to think that this reproach attaches less to the Irish medical profession than to most other sections of the community; but that it does attach in some degree is clearly shown by the incidents already alluded to.—ED. THE HOSPITAL.

